# Isoflurane Preconditioning Enhances Neuronal Tolerance to Amyloid-β Toxicity in HT-22 Cells via Mild Oxidative Signaling and Akt–Nrf2 Activation

**DOI:** 10.3390/antiox15040432

**Published:** 2026-03-30

**Authors:** Shih-Hsuan Chen, Sing-Hua Tsou, Shao-Hsing Weng, Shun-Hui Huang, Wei-Jen Chen, Chien-Ning Huang, Ching-Chi Chang, Chih-Li Lin

**Affiliations:** 1 Institute of Medicine, Chung Shan Medical University, Taichung 40201, Taiwan; chen.shih.hsuan@gmail.com (S.-H.C.); kukuking0215@gmail.com (S.-H.W.); emily113011@gmail.com (S.-H.H.); cshy049@csmu.edu.tw (C.-N.H.); 2 Department of Anesthesiology, Dalin Tzu Chi Hospital, Buddhist Tzu Chi Medical Foundation, Chiayi 62247, Taiwan; 3 Department of Medical Research, Chung Shan Medical University Hospital, Taichung 40201, Taiwan; zinminid@gmail.com; 4 Department of Biomedical Sciences, Chung Shan Medical University, Taichung 40201, Taiwan; cwj519@csmu.edu.tw; 5 Department of Internal Medicine, Division of Endocrinology and Metabolism, Chung Shan Medical University Hospital, Taichung 40201, Taiwan; 6 School of Medicine, Chung Shan Medical University, Taichung 40201, Taiwan; 7 Department of Psychiatry, Chung Shan Medical University Hospital, Taichung 40201, Taiwan

**Keywords:** isoflurane preconditioning, oxidative stress, Akt, Nrf2, Amyloid-β (Aβ), antioxidant genes

## Abstract

Isoflurane is a widely used volatile anesthetic with context-dependent effects on neuronal survival, particularly in neurodegenerative conditions. Increasing evidence suggests that brief, sublethal stress exposure can induce adaptive cellular responses through hormesis-based preconditioning mechanisms. In this study, we investigated whether isoflurane preconditioning enhances neuronal tolerance to amyloid-β (Aβ)-induced toxicity and explored the underlying redox-dependent molecular pathways. Using HT-22 murine hippocampal neuronal cells, we demonstrate that short-term exposure to low-dose isoflurane induces a delayed neuroprotective phenotype characterized by improved cell viability, reduced apoptotic signaling, and maintained mitochondrial membrane potential following Aβ challenge. Mechanistically, isoflurane preconditioning elicited a mild and transient increase in intracellular reactive oxygen species (ROS), which is critical for the activation of the PI3K/Akt signaling pathway. Pharmacological scavenging of reactive oxygen species abolished Akt phosphorylation and reduced the protective effects of preconditioning, supporting a hormetic signaling model rather than direct antioxidant action. Following Akt activation, isoflurane preconditioning promoted the inhibitory phosphorylation of glycogen synthase kinase-3β (GSK-3β), decreased Keap1 protein levels, and facilitated nuclear translocation and transcriptional activation of nuclear factor erythroid 2-related factor 2 (Nrf2). Consequently, the expression of Nrf2-regulated antioxidant genes, including heme oxygenase-1, NAD(P)H quinone dehydrogenase 1 (NQO1), superoxide dismutase 1 and 2 (SOD1/2), and catalase, was significantly upregulated. Collectively, these findings indicate that isoflurane preconditioning confers neuroprotection through hormesis-like mild oxidative signaling and coordinated activation of endogenous antioxidant defenses rather than via direct antioxidant scavenging.

## 1. Introduction

Neurons in the central nervous system (CNS) are continuously exposed to metabolic, oxidative, and proteostatic stress yet retain functional integrity for decades, highlighting the presence of intrinsic adaptive defense mechanisms [1]. The concept of hormesis describes a biphasic biological response in which low-intensity or transient stress activates endogenous protective pathways that enhance cellular resilience, whereas higher levels of the same stress become deleterious [2]. In recent years, hormesis has increasingly been adopted in neuroscience as a unifying framework to explain how neurons integrate stress signals to maintain homeostasis and resist neurodegenerative insults [3]. A well-characterized experimental manifestation of hormesis in the CNS is preconditioning, defined as a process in which brief, sublethal exposure to a stressor induces a tolerant phenotype that protects neural tissue against subsequent injury [4]. Although initially described in cardiac ischemia, preconditioning has been extensively validated in cerebral ischemia and metabolic stress models, where it significantly reduces neuronal death and improves neurological outcomes [5]. Importantly, contemporary interpretations emphasize that preconditioning does not act primarily through the acute suppression of injury pathways, but rather through coordinated reprogramming of cellular signaling, transcriptional, and metabolic networks, thereby elevating the threshold for neuronal damage [6]. At the molecular level, preconditioning engages pro-survival kinases, mitochondrial adaptive responses, and transcriptional activation of antioxidant defense systems [7]. A major conceptual advance in recent years is the recognition that reactive oxygen species (ROS) generated during preconditioning can act as signaling mediators rather than solely cytotoxic byproducts [8]. Controlled and transient ROS signaling has been shown to initiate adaptive stress responses consistent with hormetic regulation, thereby enhancing cellular resistance to subsequent oxidative challenges [9]. Among redox-sensitive transcriptional regulators, nuclear factor erythroid 2-related factor 2 (Nrf2) has emerged as a central integrator of adaptive stress responses. Under basal conditions, Nrf2 is sequestered in the cytoplasm by Kelch-like ECH-associated protein 1 (Keap1) and targeted for proteasomal degradation [10]. Upon mild oxidative stress, Nrf2 stabilizes and translocates to the nucleus to induce genes involved in antioxidant defense, detoxification, and mitochondrial protection, including heme oxygenase-1 (HO-1) and NAD(P)H quinone dehydrogenase 1 (NQO1) [11].

Among clinically used volatile anesthetics, isoflurane is particularly relevant to investigate because of its widespread clinical use and extensive experimental characterization. Isoflurane represents a compelling model anesthetic, as volatile agents are increasingly recognized as context-dependent neuromodulators whose biological effects vary according to dose, exposure timing, and cellular state [4]. Recent reviews emphasize that volatile anesthetics can exert either neuroprotective or neurotoxic effects, highlighting the inadequacy of unidirectional interpretations [6]. Isoflurane has also been central to debates linking anesthesia exposure to Alzheimer’s disease (AD)-related mechanisms [9]. Early experimental studies reported that isoflurane exposure could influence amyloid precursor protein processing and amyloid-β (Aβ) generation, raising concerns regarding possible anesthetic–AD interactions [12]. However, more recent clinical studies demonstrate no consistent long-term differences in postoperative cognition or cerebrospinal fluid AD biomarkers between isoflurane and non-volatile anesthetic regimens [13]. Complementary preclinical studies further support the context-dependent nature of isoflurane actions, showing that anesthetic exposure does not uniformly exacerbate amyloid pathology or cognitive dysfunction [14]. In contrast to acute exposure paradigms, isoflurane preconditioning has been consistently shown to induce tolerance to ischemic and metabolic insults in experimental models [5]. Recent reviews of anesthetic-induced preconditioning converge on mitochondrial preservation, redox modulation, and activation of pro-survival signaling pathways as core protective mechanisms [4]. Notably, emerging evidence also implicates Nrf2-dependent antioxidant signaling as a key mediator of isoflurane-induced neuroprotection in neuronal injury models [15]. Despite these advances, it remains unclear whether isoflurane preconditioning can confer adaptive protection against Aβ-induced oxidative and mitochondrial toxicity, which represents a central pathological feature of AD.

Building upon the concepts of hormesis-driven preconditioning and the context-dependent effects of isoflurane, the present study aims to determine whether brief, sublethal exposure to isoflurane can enhance neuronal resilience against Aβ-induced toxicity. Aβ neurotoxicity is closely associated with amplification of oxidative stress, mitochondrial dysfunction, and activation of apoptotic signaling cascades [16]. These pathological features substantially overlap with stress-responsive pathways activated during anesthetic exposure, suggesting a potential mechanistic convergence. Furthermore, recent preconditioning literature indicates that cellular protection arises not from direct antioxidant scavenging, but rather from mild and transient oxidative signaling that initiates adaptive stress responses [2]. In particular, activation of the PI3K/Akt signaling pathway has been identified as a critical mediator linking redox signaling to neuronal survival [17]. We therefore hypothesize that isoflurane preconditioning generates a controlled ROS signal that activates the Akt–GSK-3β–Keap1–Nrf2 signaling axis, leading to transcriptional activation of antioxidant gene programs and enhanced mitochondrial resilience. To test this hypothesis, we utilized an in vitro neuronal cell model to dissect the temporal dynamics and molecular mechanisms linking isoflurane preconditioning to protection against Aβ toxicity. By conceptualizing isoflurane exposure as a conditional trigger of adaptive stress responses, this study seeks to bridge the biology of anesthetic preconditioning with mechanisms relevant to neurodegeneration.

## 2. Materials and Methods

### 2.1. Reagents and Materials

The following reagents were used in this study: 3-(4,5-dimethylthiazol-2-yl)-2,5-diphenyltetrazolium bromide (MTT), 4′,6-diamidino-2-phenylindole (DAPI), 2′,7′-dichlorodihydrofluorescein diacetate (DCFH-DA), JC-1, N-acetyl-L-cysteine (NAC), LY294002, and SB216763 were purchased from Sigma-Aldrich (St. Louis, MO, USA). Amyloid-β_1_–_42_ (Aβ_1_–_42_) peptides were synthesized by LifeTein (Somerset, NJ, USA). Primary antibodies against phosphorylated Akt (Ser^473^), Akt, phosphorylated GSK-3β (Ser^9^), GSK-3β, caspase-3, and poly (ADP-ribose) polymerase (PARP) were obtained from Santa Cruz Biotechnology (Santa Cruz, CA, USA). Antibodies against superoxide dismutase 1 (SOD1), superoxide dismutase 2 (SOD2), and nuclear factor erythroid 2-related factor 2 (Nrf2) were purchased from GeneTex (Irvine, CA, USA). The antibody against heme oxygenase-1 (HO-1) was obtained from Invitrogen (Carlsbad, CA, USA). Isoflurane was purchased from Baxter Healthcare (Guayama, PR, USA).

### 2.2. Cell Culture and Isoflurane Preconditioning Protocol

The murine hippocampal neuronal cell line HT-22 was obtained from Merck (Burlington, MA, USA). Cells were maintained in Dulbecco’s Modified Eagle Medium (DMEM) supplemented with 10% fetal bovine serum, 100 U/mL penicillin, and 100 μg/mL streptomycin at 37 °C in a humidified incubator containing 5% CO_2_. Cells were routinely passaged every 2–3 days, and only cells between passages 5 and 20 (P5–P20) were used for experiments. To ensure cell culture quality, routine mycoplasma testing was performed using a commercial detection kit, and only mycoplasma-negative cultures were used in all experiments. For isoflurane preconditioning, HT-22 cells were seeded at appropriate densities according to each assay and allowed to adhere overnight. Cells were then placed in a sealed, gas-tight exposure chamber and exposed to isoflurane for 2 h at 37 °C. Isoflurane was delivered using a calibrated isoflurane vaporizer (model EZ-155; E-Z Systems Inc., Palmer, PA, USA), with the carrier gas composed of 95% air and 5% CO_2_. Control cells were treated identically but exposed to carrier gas without isoflurane. Following exposure, cells were returned to a standard humidified incubator and maintained under normal culture conditions for the indicated recovery periods. Unless otherwise specified, a 22 h recovery period was applied to allow development of delayed preconditioning responses. Where indicated, pharmacological agents were applied during the isoflurane exposure period. Pharmacological inhibitors LY294002 (20 μM, PI3K inhibitor), SB216763 (10 μM, GSK-3β inhibitor), and N-acetyl-L-cysteine (NAC, 1 mM) were dissolved in dimethyl sulfoxide (DMSO, final DMSO concentration ≤ 0.1% in all experiments) and added to the culture medium 30 min prior to isoflurane exposure and maintained throughout the exposure period. After preconditioning, cells were washed once with phosphate-buffered saline and returned to fresh culture medium for subsequent analyses.

### 2.3. Cell Viability Assessment and Aβ Preparation

Cell viability was evaluated using the MTT assay. HT-22 cells were seeded into 24-well plates at an appropriate density and allowed to adhere overnight under standard culture conditions. Following experimental treatments, MTT solution (final concentration 0.5 mg/mL) was added directly to each well, and cells were incubated at 37 °C for 4 h to allow the formation of formazan crystals. The culture medium was then removed, and the crystals were dissolved in DMSO. Absorbance was measured at 570 nm using a spectrophotometer. Cell viability was expressed as a percentage relative to the corresponding untreated control group. For amyloid-β-induced cytotoxicity experiments, synthetic Aβ_1_–_42_ peptide was used. Lyophilized Aβ_1_–_42_ was initially dissolved in hexafluoroisopropanol (HFIP) to disrupt pre-existing aggregates, aliquoted, and dried under vacuum. The peptide film was subsequently reconstituted in DMSO to prepare a concentrated stock solution and diluted in serum-free culture medium immediately before use. Under these preparation conditions, Aβ_1_–_42_ is expected to exist predominantly as low-molecular-weight species, including monomers and small oligomers, as demonstrated in our previous study [18].

### 2.4. Western Blot Analysis

Protein expression was analyzed by Western blotting. Following experimental treatments, HT-22 cells were washed twice with ice-cold phosphate-buffered saline (PBS) and lysed on ice using Gold lysis buffer containing 50 mM Tris-HCl (pH 8.0), 5 mM EDTA, 150 mM NaCl, 0.5% Nonidet P-40, 0.5 mM dithiothreitol (DTT), 1 mM phenylmethylsulfonyl fluoride (PMSF), 0.15 U/mL aprotinin, 5 μg/mL leupeptin, 1 μg/mL pepstatin, and 1 mM sodium fluoride (NaF). Cell lysates were incubated on ice for 30 min with intermittent vortexing and subsequently clarified by centrifugation at 12,000× *g* for 15 min at 4 °C. The supernatants were collected, and protein concentrations were determined using the bicinchoninic acid (BCA) protein assay (Bio-Rad, Hercules, CA, USA). Equal amounts of protein (20–30 μg per sample) were mixed with loading buffer, denatured by heating at 95 °C for 5 min, and separated by SDS–polyacrylamide gel electrophoresis (SDS–PAGE). Proteins were subsequently transferred onto polyvinylidene difluoride (PVDF) membranes. Membranes were blocked with 5% non-fat milk or 5% bovine serum albumin (BSA) in Tris-buffered saline containing 0.1% Tween-20 (TBST) for 1 h at room temperature, depending on the primary antibody used. Membranes were incubated overnight at 4 °C with primary antibodies against phosphorylated Akt (Ser^473^), total Akt, phosphorylated GSK-3β (Ser^9^), total GSK-3β, Keap1, Nrf2, cleaved poly (ADP-ribose) polymerase (PARP), cleaved caspase-3, HO-1, NQO1, SOD1, SOD2, catalase, and β-actin. After washing with TBST, membranes were incubated with appropriate horseradish peroxidase-conjugated secondary antibodies for 1 h at room temperature. Immunoreactive bands were visualized using an enhanced chemiluminescence (ECL) detection system (Millipore, Bedford, MA, USA). Densitometric analysis was performed using Quantity One softwareversion 4.6.2 (Bio-Rad, Hercules, CA, USA). Protein expression levels were normalized to β-actin or to the corresponding total protein for phosphorylated targets. Data were expressed as fold changes relative to the control group and represent the mean ± standard error of the mean from at least three independent experiments.

### 2.5. Measurement of Intracellular Reactive Oxygen Species by DCFH-DA Staining

Intracellular ROS levels were assessed using the fluorescent probe DCFH-DA. HT-22 cells were seeded onto coverslips or culture plates at appropriate densities and subjected to experimental treatments as indicated. Following isoflurane preconditioning and subsequent recovery periods, cells were incubated with DCFH-DA (10 μM) diluted in serum-free culture medium for 30 min at 37 °C in the dark. After incubation, cells were gently washed three times with PBS to remove excess probe. Intracellular fluorescence, reflecting ROS-dependent oxidation of DCFH to fluorescent dichlorofluorescein (DCF), was immediately visualized using a fluorescence microscope under identical exposure settings for all experimental groups. All experiments were repeated independently at least three times to ensure reproducibility.

### 2.6. Assessment of Mitochondrial Membrane Potential by JC-1 Staining

Mitochondrial membrane potential (ΔΨm) was assessed using the fluorescent dye JC-1, which exhibits potential-dependent accumulation in mitochondria. HT-22 cells were seeded onto coverslips or culture plates and subjected to experimental treatments as indicated. Following isoflurane preconditioning and subsequent recovery periods, cells were incubated with JC-1 working solution (final concentration 5 μM) in serum-free culture medium for 30 min at 37 °C in the dark. After incubation, cells were gently washed with PBS to remove excess dye. Fluorescence signals were immediately visualized using a fluorescence microscope. JC-1 aggregates, indicative of polarized mitochondria, were detected as red fluorescence, whereas JC-1 monomers, indicative of mitochondrial depolarization, were detected as green fluorescence. Images were acquired under identical exposure settings for all experimental groups. For quantitative analysis, the ratio of red to green fluorescence intensity was calculated to evaluate changes in mitochondrial membrane potential. Fluorescence intensities were quantified using Image pro plus 6.0 (Media Cybernetics, Rockville, MD, USA). Representative images and quantitative analyses were obtained from randomly selected fields in at least three independent experiments.

### 2.7. Quantitative Analysis of Gene Expression by Reverse Transcription–Quantitative PCR (RT–qPCR)

Total RNA was extracted from HT-22 cells using the RNeasy Kit (Qiagen, Germantown, MD, USA) according to the manufacturer’s instructions. RNA concentration and purity were determined spectrophotometrically to ensure adequate quality for downstream applications. For reverse transcription, 1 μg of total RNA was converted into complementary DNA (cDNA) using a commercial reverse transcription kit and a TProfessional Thermocycler (Biometra, Göttingen, Germany) under optimized reaction conditions. Quantitative PCR (qPCR) was performed using Power SYBR Green PCR Master Mix on an ABI 7300 Sequence Detection System (Applied Biosystems, Foster City, CA, USA). Each cDNA sample was analyzed in triplicate. The thermal cycling conditions consisted of an initial denaturation step at 95 °C for 10 min, followed by 40 amplification cycles of 95 °C for 15 s and 60 °C for 1 min. A melt curve analysis was performed at the end of each run to confirm the specificity of amplification. The expression levels of antioxidant and stress-responsive genes, including HO-1 (*Hmox1*), NQO1 (*Nqo1*), SOD1 (*Sod1*), SOD2 (*Sod2*), and catalase (*Cat*), were quantified. Glyceraldehyde-3-phosphate dehydrogenase (*Gapdh*) was used as an internal reference gene. Relative mRNA expression levels were calculated using the 2^−ΔΔCt^ method and normalized to GAPDH. Primer sequences for all target and reference genes are provided in Table 1. Data analysis was performed using Sequence Detection System software (v3.0, Applied Biosystems, Foster City, CA, USA).

### 2.8. Immunocytochemical Analysis

Immunocytochemical analysis was performed to examine the subcellular localization of Nrf2. HT-22 cells were seeded onto sterile glass coverslips and subjected to experimental treatments as indicated. At the designated time points following isoflurane preconditioning, cells were washed with PBS and fixed with 4% paraformaldehyde for 15 min at room temperature. Paraformaldehyde is a polymer that, upon exposure to water, dissociates into formaldehyde, which is the active molecule responsible for fixation. After fixation, cells were permeabilized with 0.1% Triton X-100 in PBS for 10 min and then blocked with 5% BSA in PBS for 1 h at room temperature to reduce nonspecific binding. Cells were subsequently incubated overnight at 4 °C with a primary antibody against Nrf2 diluted in blocking solution. Following primary antibody incubation, cells were washed with PBS and incubated with the appropriate fluorescent dye-conjugated secondary antibody for 1 h at room temperature in the dark. Nuclei were counterstained with DAPI. Coverslips were mounted using an antifade mounting medium. Fluorescence images were acquired using a fluorescence microscope under identical acquisition settings for all experimental groups. Nrf2 nuclear localization was qualitatively assessed by examining the overlap between Nrf2 immunofluorescence and DAPI nuclear staining. Representative images were obtained from randomly selected fields in at least three independent experiments.

### 2.9. Nrf2-Dependent Promoter Activity Assay

Nrf2 transcriptional activity was assessed using a dual-luciferase reporter assay driven by antioxidant response elements (AREs). HT-22 cells were seeded into 24-well plates and transfected with an ARE luciferase reporter vector Lipofectamine 3000 (Thermo Fisher Scientific, Waltham, MA, USA) according to the manufacturer’s instructions. The reporter construct was obtained from the ARE Reporter Kit (AMS.60514, Amsbio, Abingdon, UK), which contains a firefly luciferase gene under the control of multimerized ARE sequences upstream of a minimal promoter, together with a constitutively expressed Renilla luciferase vector as an internal control for transfection efficiency. After transfection, cells were allowed to recover for 24 h and subsequently subjected to isoflurane preconditioning and pharmacological treatments as indicated. At the designated time points following treatment, cells were lysed, and luciferase activities were measured using a dual-luciferase assay system according to the manufacturer’s instructions. Luminescence signals for firefly and Renilla luciferase were sequentially detected using a multi-mode microplate reader (iD5, Molecular Devices, San Jose, CA, USA). Firefly luciferase activity was normalized to Renilla luciferase activity to control for transfection efficiency. In selected experiments, a non-inducible luciferase reporter vector provided in the kit, containing a minimal promoter without ARE sequences, was used as a negative control to assess background luciferase activity and confirm pathway specificity. Normalized luciferase activity was expressed as fold change relative to the corresponding control group. All experiments were performed in triplicate and repeated independently at least three times.

### 2.10. Statistical Analysis

All quantitative data are presented as the mean ± standard error of the mean (SEM) from at least three independent experiments. Statistical analyses were performed using GraphPad Prism software (version 10). Comparisons between two groups were performed using an unpaired Student’s *t*-test, whereas comparisons among multiple groups were analyzed by one-way analysis of variance (ANOVA) followed by Tukey’s post hoc test for multiple comparisons. A value of *p* < 0.05 was considered statistically significant.

## 3. Results

### 3.1. Isoflurane Preconditioning Enhances Neuronal Tolerance to Aβ-Induced Cytotoxicity in HT-22 Cells

**Figure 1:**
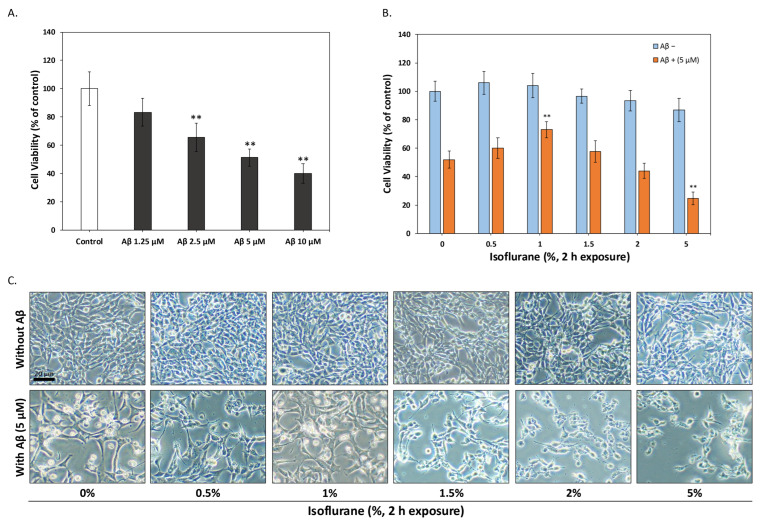
Isoflurane preconditioning protects HT-22 hippocampal neuronal cells from Aβ_1–42_–induced cytotoxicity. (**A**) HT-22 cells were treated with increasing concentrations of Aβ_1–42_ (1.25–10 μM) for 24 h, and cell viability was assessed by MTT assay. Aβ induced a dose-dependent decrease in cell viability, and 5 μM was selected as the experimental concentration for subsequent experiments. (**B**) HT-22 cells were exposed to isoflurane (0–5%) for 2 h, followed by a 22 h recovery period, and then challenged with 5 μM Aβ for 24 h. Isoflurane preconditioning at 0.5–1% significantly attenuated Aβ-induced cytotoxicity, with maximal protection observed at 1% isoflurane. (**C**) Representative phase-contrast micrographs of HT-22 cells with or without Aβ exposure following isoflurane preconditioning. Scale bar, 20 μm. Data are presented as mean ± SEM. ** *p* < 0.01 compared with control groups.

To establish an in vitro model of Aβ-induced neurotoxicity, HT-22 hippocampal neuronal cells were exposed to increasing concentrations of Aβ_1–42_ for 24 h, and cell viability was assessed using the MTT assay. As shown in Figure 1A, Aβ induced a dose-dependent decrease in cell viability, with significant cytotoxicity observed at concentrations ≥ 2.5 μM. Based on these results, 5 μM Aβ, which reduced cell viability by approximately 50%, was selected as the working concentration for subsequent experiments. Next, we examined whether brief isoflurane exposure could induce a preconditioning response that alters neuronal susceptibility to Aβ toxicity. HT-22 cells were exposed to different concentrations of isoflurane (0–5%) for 2 h, followed by a 22 h recovery period under normal culture conditions. Cells were then challenged with 5 μM Aβ for an additional 24 h, and cell viability was measured. As shown in Figure 1B, isoflurane preconditioning significantly altered Aβ-induced cytotoxicity in a concentration-dependent manner. Preconditioning with 0.5–1% isoflurane markedly attenuated Aβ-induced cell death, with 1% isoflurane providing the most robust protective effect. In contrast, higher concentrations of isoflurane (≥2%) failed to confer protection and were associated with reduced cell viability, suggesting a narrow therapeutic window for effective preconditioning. Consistent with the MTT results, phase-contrast microscopy revealed clear morphological differences between treatment groups (Figure 1C). In the absence of Aβ, HT-22 cells maintained a spindle-shaped morphology across lower isoflurane concentrations, whereas higher concentrations induced visible cellular stress. Following Aβ exposure, cells exhibited pronounced rounding, detachment, and loss of neurite-like cellular extensions. Notably, cells preconditioned with 1% isoflurane displayed improved cell attachment and preserved morphology compared with Aβ-treated controls, further supporting a protective effect of isoflurane preconditioning. Together, these results identify 1% isoflurane exposure for 2 h as an optimal preconditioning condition that enhances neuronal tolerance to Aβ-induced toxicity in HT-22 cells.

### 3.2. Isoflurane Preconditioning Induces Transient Akt Activation Required for Protection Against Aβ-Induced Apoptosis

**Figure 2:**
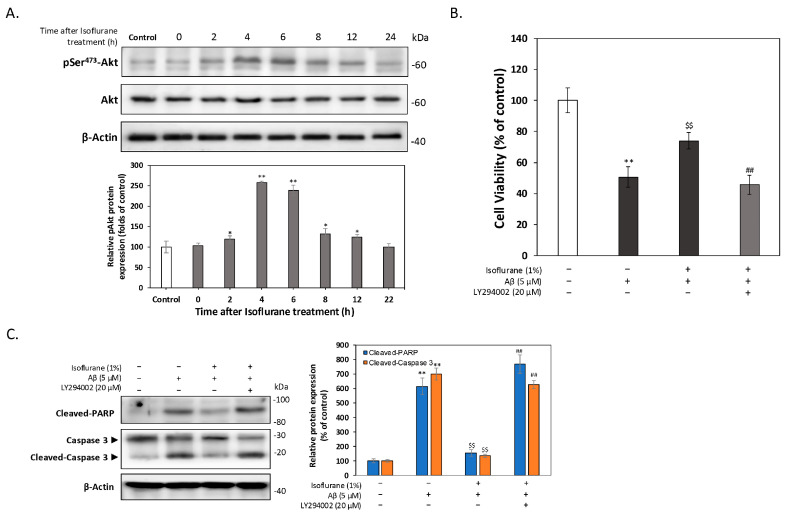
Isoflurane preconditioning induces transient Akt activation and protects against Aβ-induced apoptosis via PI3K/Akt signaling. (**A**) HT-22 cells were exposed to 1% isoflurane for 2 h, and phosphorylated Akt (Ser^473^), total Akt, and β-actin were analyzed by Western blot at the indicated time points during the recovery period. Quantification of band intensity shows a transient increase in Akt phosphorylation, peaking at 4–6 h after isoflurane exposure. (**B**) Cell viability was assessed by MTT assay following isoflurane preconditioning, recovery, and subsequent challenge with 5 μM Aβ for 24 h. The PI3K inhibitor LY294002 (20 μM) was applied during isoflurane exposure. Inhibition of PI3K significantly attenuated the protective effect of isoflurane preconditioning. (**C**) Representative Western blots and densitometric quantification of cleaved PARP and cleaved caspase-3 following indicated treatments. Isoflurane preconditioning reduced Aβ-induced activation of apoptotic markers, whereas LY294002 reversed this effect. Data are presented as mean ± SEM. * *p* < 0.05 and ** *p* < 0.01 compared with control; $$ *p* < 0.01 compared with Aβ-treated group; ## *p* < 0.01 compared with isoflurane-preconditioned group. the symbols ‘+’ and ‘−’ denote the presence and absence, respectively, of the indicated treatment conditions.

To determine whether isoflurane preconditioning activates the PI3K/Akt survival pathway, we first examined the temporal profile of Akt phosphorylation following isoflurane exposure. HT-22 cells were exposed to 1% isoflurane for 2 h, and phosphorylated Akt (Ser^473^) levels were analyzed at indicated time points during the recovery period. As shown in Figure 2A, isoflurane preconditioning induced a time-dependent increase in Akt phosphorylation without altering total Akt protein levels. Akt phosphorylation became detectable approximately 2 h after isoflurane exposure, reached a peak at 4–6 h, and gradually declined thereafter, returning toward baseline by 24 h. These findings indicate that isoflurane preconditioning triggers a transient activation of Akt signaling rather than sustained pathway stimulation. We next assessed whether Akt activation is required for the neuroprotective effect of isoflurane preconditioning against Aβ toxicity. HT-22 cells were subjected to isoflurane preconditioning followed by a 22 h recovery period and then challenged with 5 μM Aβ for 24 h. Cell viability was evaluated using the MTT assay. As shown in Figure 2B, isoflurane preconditioning significantly attenuated Aβ-induced cytotoxicity. Importantly, co-treatment with the PI3K inhibitor LY294002 (20 μM) during the isoflurane exposure phase markedly reduced this protective effect, indicating that PI3K/Akt signaling is required for isoflurane preconditioning-mediated neuroprotection. To further determine whether Akt activation mediates suppression of Aβ-induced apoptosis, we examined the expression of two well-established apoptotic markers, cleaved PARP and cleaved caspase-3. As shown in Figure 2C, Aβ treatment markedly increased the levels of cleaved PARP and cleaved caspase-3, consistent with apoptotic cell death. Isoflurane preconditioning significantly reduced the activation of both apoptotic markers. In contrast, inhibition of PI3K signaling with LY294002 abolished the anti-apoptotic effects of isoflurane preconditioning, restoring apoptotic marker levels to those observed in Aβ-treated cells.

### 3.3. Isoflurane Preconditioning Engages Akt-Dependent Redox and Mitochondrial Protection

**Figure 3:**
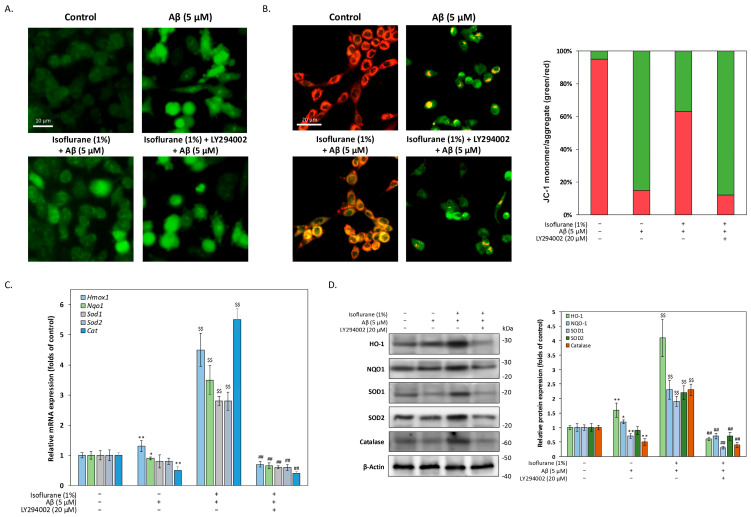
Isoflurane preconditioning attenuates oxidative stress, preserves mitochondrial integrity, and induces antioxidant gene expression through PI3K/Akt signaling. (**A**) Intracellular ROS levels were assessed using DCFH-DA fluorescence staining following isoflurane preconditioning, recovery, and subsequent Aβ_1–42_ challenge. Isoflurane preconditioning partially reduced Aβ-induced ROS accumulation, whereas PI3K inhibition with LY294002 attenuated this effect. (**B**) Mitochondrial membrane potential was evaluated using JC-1 staining. Aβ exposure increased the proportion of JC-1 monomers (green fluorescence), indicating mitochondrial membrane depolarization. Isoflurane preconditioning preserved mitochondrial membrane potential, while LY294002 diminished this protective effect. Quantification of JC-1 aggregate-to-monomer fluorescence ratios is shown on the right. The bar graph shows the ratio of JC-1 monomer/aggregate (green/red). (**C**) Quantitative PCR analysis of antioxidant gene expression following indicated treatments. Isoflurane preconditioning significantly increased mRNA levels of the antioxidant genes HO-1, NQO1, SOD1, SOD2, and catalase, an effect abolished by PI3K inhibition. (**D**) Representative Western blots and densitometric quantification confirming increased protein expression of antioxidant enzymes following isoflurane preconditioning, which was blocked by LY294002. Data are presented as mean ± SEM. * *p* < 0.05 and ** *p* < 0.01 compared with control; $$ *p* < 0.01 compared with Aβ-treated group; ## *p* < 0.01 compared with isoflurane-preconditioned group. the symbols ‘+’ and ‘−’ denote the presence and absence, respectively, of the indicated treatment conditions.

To determine whether isoflurane preconditioning modulates oxidative stress responses to Aβ, intracellular ROS levels were assessed using DCFH-DA fluorescence staining. HT-22 cells were exposed to 1% isoflurane for 2 h, followed by a 22 h recovery period under normal culture conditions, and subsequently challenged with 5 μM Aβ for 24 h. As shown in Figure 3A, Aβ treatment markedly increased intracellular ROS accumulation compared with control cells. Isoflurane preconditioning significantly attenuated Aβ-induced ROS accumulation, although ROS levels remained higher than those observed in untreated control cells, suggesting an enhanced capacity to buffer oxidative stress rather than complete suppression of ROS generation. Importantly, inhibition of PI3K signaling with LY294002 during the isoflurane exposure phase significantly diminished this effect, indicating that PI3K/Akt activation contributes to the modulation of oxidative stress following preconditioning. Because mitochondria represent a major intracellular source of ROS and are highly susceptible to Aβ-induced damage, we next examined mitochondrial membrane potential using JC-1 staining. As shown in Figure 3B, Aβ exposure resulted in a pronounced loss of mitochondrial membrane potential, as evidenced by an increased proportion of JC-1 monomers (green fluorescence) and a concomitant reduction in JC-1 aggregates (red fluorescence). Isoflurane preconditioning markedly reversed this effect, indicating preservation of mitochondrial membrane potential and bioenergetic integrity. In contrast, co-treatment with LY294002 substantially attenuated the mitochondrial protective effect of isoflurane preconditioning, further supporting a role for PI3K/Akt signaling in maintaining mitochondrial integrity. To further determine whether the observed redox and mitochondrial protection was associated with activation of antioxidant gene programs, we examined the expression of Nrf2-regulated genes. We then investigated whether isoflurane preconditioning induces an adaptive antioxidant transcriptional response. Quantitative PCR analysis revealed that isoflurane preconditioning significantly upregulated the mRNA expression levels of multiple antioxidant and cytoprotective genes, including HO-1, NQO1, SOD1, SOD2, and catalase (Figure 3C). Notably, this transcriptional induction was abolished by PI3K inhibition with LY294002, indicating that activation of PI3K/Akt signaling is required for the transcriptional upregulation of antioxidant defense genes following isoflurane preconditioning. Consistent with the transcriptional data, Western blot analysis confirmed that isoflurane preconditioning increased the protein expression levels of HO-1, NQO1, SOD1, SOD2, and catalase (Figure 3D). Inhibition of PI3K signaling with LY294002 effectively blocked the upregulation of these antioxidant proteins, further validating that PI3K/Akt signaling mediates the adaptive antioxidant response induced by isoflurane preconditioning. These findings indicate that isoflurane preconditioning confers Akt-dependent protection against Aβ-induced oxidative and mitochondrial damage through coordinated activation of antioxidant defense pathways.

### 3.4. Isoflurane Preconditioning Activates the Akt–GSK-3β–Keap1–Nrf2 Axis to Induce an Adaptive Antioxidant Transcriptional Program

**Figure 4:**
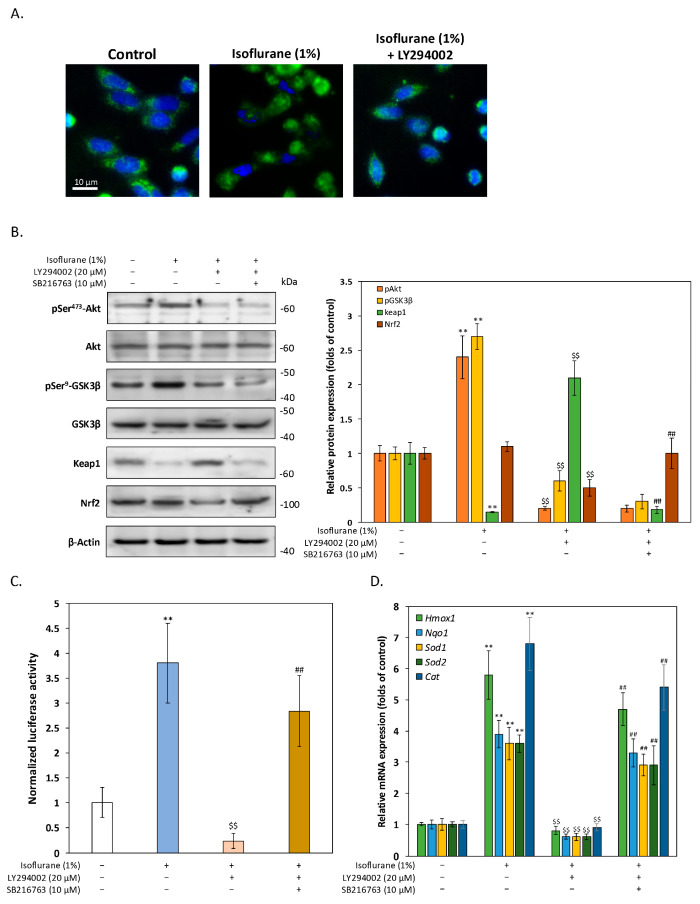
Isoflurane preconditioning activates the Akt–GSK-3β–Keap1–Nrf2 signaling axis and induces antioxidant gene expression. (**A**) Representative immunofluorescence images showing Nrf2 localization (green) and nuclei stained with DAPI (blue) at 4 h after isoflurane preconditioning. Isoflurane preconditioning induced Nrf2 nuclear translocation, which was abolished by PI3K inhibition with LY294002. Scale bar = 10 μm. (**B**) Western blot analysis of phosphorylated Akt (Ser^473^), phosphorylated GSK-3β (Ser^9^), Keap1, and Nrf2 at 4 h after isoflurane exposure. Densitometric analysis demonstrates that PI3K inhibition suppresses Akt and GSK-3β phosphorylation and prevents Keap1 reduction and Nrf2 activation, whereas GSK-3β inhibition with SB216763 restores Nrf2 activation. (**C**) ARE-driven luciferase reporter assay showing increased Nrf2 transcriptional activity following isoflurane preconditioning. LY294002 inhibited this effect, while SB216763 rescued Nrf2 activity. (**D**) Quantitative PCR analysis of Nrf2 downstream antioxidant target genes (HO-1, NQO1, SOD1, SOD2, catalase) at 4 h after isoflurane exposure. Isoflurane-induced gene expression was blocked by PI3K inhibition and restored by GSK-3β inhibition. Data are presented as mean ± SEM. ** *p* < 0.01 compared with control; $$ *p* < 0.01 compared with isoflurane-preconditioned group; ## *p* < 0.01 compared with LY294002-treated group. the symbols ‘+’ and ‘−’ denote the presence and absence, respectively, of the indicated treatment conditions.

To determine whether isoflurane preconditioning activates Nrf2 signaling, we first examined the subcellular localization of Nrf2 by immunofluorescence staining. HT-22 cells were exposed to 1% isoflurane for 2 h, and Nrf2 localization was analyzed 4 h after the completion of isoflurane exposure. As shown in Figure 4A, Nrf2 was predominantly localized in the cytoplasm under basal conditions. Isoflurane preconditioning markedly promoted Nrf2 nuclear translocation, consistent with activation of Nrf2 transcriptional function. In contrast, inhibition of PI3K signaling with LY294002 abolished isoflurane-induced Nrf2 nuclear accumulation, indicating that PI3K/Akt signaling is required for Nrf2 activation following preconditioning. We next investigated the upstream signaling events linking isoflurane preconditioning to Nrf2 activation. Western blot analysis revealed that isoflurane preconditioning significantly increased phosphorylation of Akt (Ser^473^) and its downstream substrate GSK-3β at the inhibitory Ser^9^ site at 4 h after exposure (Figure 4B). Concomitantly, Keap1 protein levels were reduced, accompanied by an increase in Nrf2 expression. Pharmacological inhibition of PI3K with LY294002 suppressed Akt and GSK-3β phosphorylation, restored Keap1 levels, and prevented Nrf2 accumulation. Notably, co-treatment with the selective GSK-3β inhibitor SB216763 restored Keap1 reduction and Nrf2 expression despite PI3K inhibition, supporting the conclusion that suppression of GSK-3β activity downstream of Akt is sufficient to facilitate Nrf2 activation. To further confirm functional activation of Nrf2, Nrf2-dependent transcriptional activity was assessed using a luciferase reporter assay. As shown in Figure 4C, isoflurane preconditioning significantly increased ARE-driven luciferase activity. This induction was markedly attenuated by LY294002, whereas co-treatment with SB216763 restored Nrf2 transcriptional activity in the presence of PI3K inhibition. These results further support a model in which Akt-mediated inhibition of GSK-3β is required for Nrf2 transcriptional activation following isoflurane preconditioning. These findings prompted us to determine whether Nrf2 activation resulted in increased expression of downstream antioxidant genes. Quantitative PCR analysis demonstrated that isoflurane preconditioning robustly upregulated the mRNA expression of multiple Nrf2 target genes, including HO-1, NQO1, SOD1, SOD2, and catalase, at 4 h after exposure (Figure 4D). Inhibition of PI3K signaling with LY294002 abolished this transcriptional induction, whereas co-treatment with SB216763 significantly restored the expression of these antioxidant genes. Collectively, these findings indicate that isoflurane preconditioning activates an adaptive antioxidant transcriptional program through the Akt–GSK-3β–Keap1–Nrf2 signaling axis, linking upstream Akt signaling to downstream antioxidant gene induction.

### 3.5. Mild Oxidative Signaling During Isoflurane Preconditioning Is Required for Akt Activation and Neuroprotection

**Figure 5:**
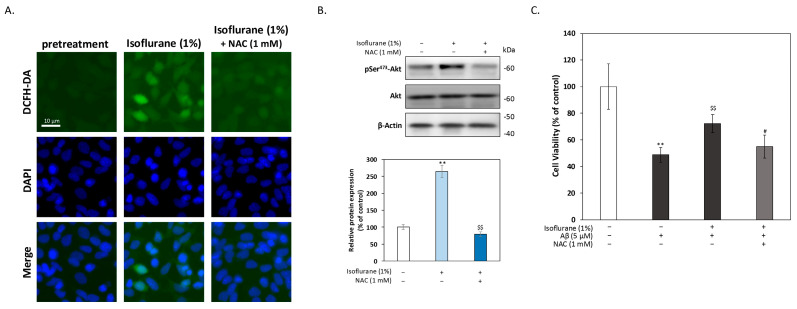
Mild oxidative signaling during isoflurane preconditioning is required for Akt activation and neuroprotection. (**A**) Intracellular ROS levels were assessed using DCFH-DA fluorescence staining immediately after 1% isoflurane exposure. Isoflurane preconditioning induced a modest increase in ROS, which was abolished by co-treatment with N-acetylcysteine (NAC, 1 mM). Nuclei were counterstained with DAPI (blue). Scale bar = 10 μm. (**B**) Western blot analysis of phosphorylated Akt (Ser^473^) and total Akt at 4 h after isoflurane exposure. NAC treatment during isoflurane exposure significantly attenuated Akt phosphorylation. Densitometric quantification is shown below. (**C**) Cell viability was assessed by MTT assay following isoflurane preconditioning with or without NAC, recovery, and subsequent challenge with 5 μM Aβ for 24 h. NAC significantly reduced the protective effect of isoflurane preconditioning. Data are presented as mean ± SEM. ** *p* < 0.01 compared with control; $$ *p* < 0.01 compared with Aβ-treated group; # *p* < 0.05 compared with isoflurane-preconditioned group. the symbols ‘+’ and ‘−’ denote the presence and absence, respectively, of the indicated treatment conditions.

To determine whether mild oxidative signaling serves as an upstream trigger for Akt activation during isoflurane preconditioning, intracellular reactive oxygen species (ROS) levels were examined using DCFH-DA fluorescence staining. HT-22 cells were exposed to 1% isoflurane for 2 h, and intracellular ROS levels were assessed immediately after exposure. As shown in Figure 5A, isoflurane preconditioning induced a modest increase in intracellular ROS compared with pretreatment controls. Notably, co-treatment with the ROS scavenger NAC (1 mM) during the isoflurane exposure phase effectively abolished this mild ROS accumulation, indicating that isoflurane preconditioning induces a transient and scavenger-sensitive oxidative signal. We next examined whether this mild oxidative signal is required for activation of the PI3K/Akt pathway. Western blot analysis revealed that isoflurane preconditioning significantly increased Akt phosphorylation at 4 h after exposure (Figure 5B). Importantly, NAC treatment during isoflurane exposure markedly attenuated Akt phosphorylation without altering total Akt levels, suggesting that mild ROS generation is required for Akt activation following isoflurane preconditioning. Finally, we examined the functional relevance of ROS-dependent Akt activation in mediating neuroprotection against Aβ toxicity. HT-22 cells were subjected to isoflurane preconditioning in the presence or absence of NAC, followed by a recovery period and subsequent challenge with 5 μM Aβ for 24 h. Cell viability was assessed using the MTT assay. As shown in Figure 5C, isoflurane preconditioning significantly improved cell viability following Aβ exposure. In contrast, NAC treatment during the preconditioning phase significantly diminished the protective effect of isoflurane preconditioning. These findings indicate that mild oxidative signaling during isoflurane exposure acts as a critical upstream trigger linking isoflurane preconditioning to Akt activation and subsequent neuroprotection, consistent with the concept of hormesis and adaptive stress responses.

## 4. Discussion

The present study demonstrates that brief, sublethal isoflurane exposure induces a preconditioning effect that enhances neuronal tolerance to Aβ-induced toxicity through a coordinated adaptive signaling cascade. Specifically, our findings support a model in which isoflurane preconditioning generates a mild and transient oxidative signal that activates the PI3K/Akt pathway, leading to inhibition of GSK-3β activity, functional release of Nrf2 from Keap1-mediated repression, and subsequent induction of antioxidant and cytoprotective gene programs. This adaptive response preserves mitochondrial integrity, limits oxidative stress accumulation, and attenuates apoptotic signaling following Aβ challenge, collectively enhancing neuronal resilience. Our data thus integrate preconditioning biology with Aβ-related neurotoxicity through a hormesis-based framework of stress adaptation [19]. ROS has been reported to activate the PI3K/Akt pathway through redox-dependent inhibition of the phosphatase PTEN, a key negative regulator of PI3K signaling. Oxidative modification of PTEN suppresses its phosphatase activity, resulting in accumulation of phosphatidylinositol (3,4,5)-trisphosphate (PIP3) and subsequent Akt activation [20,21]. This mechanism is consistent with our findings, in which a mild increase in ROS during isoflurane preconditioning triggers Akt activation and downstream neuroprotection. Recent studies further highlight that the PI3K/Akt/Nrf2 signaling axis represents a central integrative pathway in neuroprotection across Alzheimer’s disease, ischemic injury, and perioperative neurocognitive disorders, reinforcing the biological relevance of our findings [4,22]. A key conceptual implication of this work is the reframing of isoflurane as a conditional modulator of neuronal fate rather than an intrinsically neuroprotective or neurotoxic agent. Volatile anesthetics have long been associated with seemingly contradictory effects on the brain, which are increasingly understood to depend on exposure timing, dosage, and cellular vulnerability. Recent reviews emphasize that anesthetic-induced neuroprotection follows a hormetic paradigm, where low-intensity stress activates adaptive defense pathways, whereas excessive exposure leads to injury [4,8]. Our findings are consistent with this framework, demonstrating that brief isoflurane exposure engages endogenous defense mechanisms that shift neuronal responses toward tolerance rather than degeneration.

A central feature of this adaptive process is mild oxidative signaling, which our data identify as a necessary upstream trigger for Akt activation. Rather than acting solely as a damaging agent, ROS in this context functions as a signaling mediator that initiates protective pathways. This interpretation is supported by the observation that ROS scavenging abolishes Akt activation and attenuates neuroprotection. This concept aligns with emerging redox biology, which recognizes ROS as dual-function signaling molecules that regulate stress adaptation and mitochondrial homeostasis [23,24]. Downstream of ROS signaling, our study identifies the PI3K/Akt pathway as a critical mediator linking preconditioning to transcriptional reprogramming. Akt activation has been widely implicated in neuronal survival [22], and our findings demonstrate that inhibition of PI3K abolishes both antioxidant gene induction and cytoprotection. These results place Akt at the center of the preconditioning response. Our data showing that GSK-3β inhibition restores Nrf2 activation even under PI3K blockade strengthens the causal structure of the Akt–GSK-3β–Nrf2 axis.

A notable mechanistic insight from this study is that inhibition of GSK-3β serves as a key downstream event linking Akt activation to Nrf2 signaling. Inhibitory phosphorylation of GSK-3β has been shown to facilitate Nrf2 stabilization by suppressing both Keap1-dependent and Keap1-independent degradation pathways, thereby promoting nuclear accumulation of Nrf2 [17]. Activation of Nrf2-dependent antioxidant gene programs represents a major effector mechanism in this model. Nrf2 is widely recognized as a master regulator of cellular stress adaptation, coordinating antioxidant defense and mitochondrial function. Recent studies demonstrate that pharmacological activation of Nrf2 confers neuroprotection in multiple disease models, including ischemia and neurodegeneration, highlighting its therapeutic potential [24,25]. Our findings extend this concept by positioning Nrf2 as a key mediator of anesthetic-induced preconditioning in an Aβ-relevant context. Importantly, the relevance of these findings extends beyond intracellular signaling mechanisms to broader strategies for neurodegenerative disease. Aβ toxicity is closely associated with oxidative stress and mitochondrial dysfunction, and enhancing endogenous stress-response capacity may represent a complementary therapeutic strategy. This concept is consistent with emerging “preconditioning-mimetic” approaches, in which pharmacological agents reproduce adaptive stress responses without requiring the original stimulus.

However, the clinical translational implications of the present findings should be interpreted with caution. Isoflurane is an inhalational anesthetic administered in the context of surgery, and its prophylactic use to prevent AD is not clinically feasible. Rather than proposing direct anesthetic intervention, our findings should be interpreted as identifying a conserved adaptive signaling pathway that may be targeted by alternative pharmacological or physiological strategies. Although mechanistically distinct from anesthetic preconditioning, interestingly recent large-scale epidemiological analyses have shown that certain systemic interventions, such as vaccination, may be associated with reduced dementia risk [26], suggesting that modulation of systemic or stress-response pathways can influence neurodegenerative outcomes. While mechanistically distinct, such findings support the broader concept that enhancing resilience pathways may represent a viable strategy for modifying disease risk. From a perioperative perspective, our findings may have greater relevance in acute settings rather than long-term disease prevention. Modern anesthesia practice increasingly emphasizes maintaining an “appropriate” depth of anesthesia using monitoring tools such as the bispectral index (BIS). It is conceivable that maintaining optimal anesthetic depth may be associated with modulation of neurological outcomes by engaging adaptive pathways similar to those identified in this study. In this context, anesthetics may potentially act not only as agents of unconsciousness but also as modulators of neurobiological stress responses. This perspective is further supported by recent clinical trials such as the REGAIN study [27], which highlight the importance of anesthetic strategy in influencing functional and neurological outcomes. Although direct neuroprotective effects remain inconclusive, these findings suggest that anesthetic management may play a broader role in patient recovery than previously appreciated. Importantly, this study does not advocate the clinical use of isoflurane as a preventive intervention for AD, but rather highlights a mechanistic framework that may guide future development of targeted neuroprotective strategies.

Isoflurane is routinely administered at clinical MAC (minimum alveolar concentration) concentrations of 1.2–1.5% during general anesthesia, which is comparable to the effective preconditioning dose of 1% for 2 h used in our experimental model and therefore suggests potential, although indirect, perioperative relevance [28]. However, translation from HT-22 cell models to human pathology remains limited by species differences, the complexity of the in vivo brain environment, and patient-specific factors such as aging and comorbidities. Several limitations should be acknowledged. First, this study is based on an in vitro neuronal model and acute Aβ exposure, which may not fully recapitulate chronic neurodegenerative processes. Second, although we establish a mechanistic signaling cascade, long-term adaptive responses such as epigenetic reprogramming were not examined. Third, in vivo validation is required to confirm translational relevance. In conclusion, this study provides mechanistic evidence that isoflurane preconditioning induces a hormesis-like adaptive response in neuronal cells that enhances neuronal tolerance to Aβ-induced toxicity through a ROS–Akt–GSK-3β–Nrf2 signaling axis. By reframing isoflurane as a tool to uncover endogenous neuroprotective mechanisms rather than a direct therapeutic agent, our findings provide a conceptual framework for the development of preconditioning-based or pathway-targeted strategies to mitigate neurodegenerative vulnerability.

## Figures and Tables

**Table 1:** Primer sequence of different genes for qPCR analysis.

Genes	Forward (5′-3′)	Reverse (5′-3′)	Accession Number
*Hmox1*	AGGCTAAGACCGCCTTCCT	AAAGCCCTACAGCAACTGTC	NM_010442
*Nqo1*	AGGCTGGTTTGAGCGAGTTA	ATTGAATTCGGGCGTCTGCT	NM_008706
*Sod1*	AACCAGTTGTGTTGTCAGGAC	CCACCATGTTTCTTAGAGTGAGG	NM_011434
*Sod2*	CAGACCTGCCTTACGACTATGG	CTCGGTGGCGTTGAGATTGTT	NM_013671
*Cat*	AGCGACCAGATGAAGCAGTG	TCCGCTCTCTGTCAAAGTGTG	NM_009804
*Gapdh*	AGGTCGGTGTGAACGGATTTG	TGTAGACCATGTAGTTGAGGTCA	NM_001289726

## Data Availability

The original contributions presented in this study are included in the article. Further inquiries can be directed to the corresponding author(s).

## Abbreviations

The following abbreviations are used in this manuscript:

Aβ	amyloid-β
AD	Alzheimer’s disease
ARE	antioxidant response element
CNS	central nervous system
DAPI	4′,6-diamidino-2-phenylindole
DCFH-DA	2′,7′-dichlorodihydrofluorescein diacetate
DMSO	dimethyl sulfoxide
GSK-3β	glycogen synthase kinase-3β
HFIP	hexafluoroisopropanol
HO-1	heme oxygenase-1
Keap1	Kelch-like ECH-associated protein 1
MAC	minimum alveolar concentration
MTT	3-(4,5-dimethylthiazol-2-yl)-2,5-diphenyltetrazolium bromide
NAC	N-acetyl-L-cysteine
NQO1	NAD(P)H quinone dehydrogenase 1
Nrf2	nuclear factor erythroid 2-related factor 2
PARP	poly (ADP-ribose) polymerase
ROS	reactive oxygen species
SOD1/2	superoxide dismutase 1 and 2
